# Beyond the cerebellum: perivascular space burden in spinocerebellar ataxia type 3 extends to multiple brain regions

**DOI:** 10.1093/braincomms/fcaf118

**Published:** 2025-03-27

**Authors:** Yonghua Huang, Zhiming Zhen, Lihua Deng, Peiling Ou, Linfeng Shi, Feng Shi, Rui Hua, Jiaojiao Wu, Wei Chen, Ru Wen, Jian Wang, Chen Liu

**Affiliations:** 7T Magnetic Resonance Imaging Translational Medical Center, Southwest Hospital, Army Medical University (Third Military Medical University), Chongqing 400038, China; Department of Radiology, Southwest Hospital, Army Medical University (Third Military Medical University), Chongqing 400038, China; Department of Radiology, The 940th Hospital of the Joint Logistics Support Force of the Chinese People's Liberation Army, Lanzhou 730050, China; 7T Magnetic Resonance Imaging Translational Medical Center, Southwest Hospital, Army Medical University (Third Military Medical University), Chongqing 400038, China; Department of Radiology, Southwest Hospital, Army Medical University (Third Military Medical University), Chongqing 400038, China; 7T Magnetic Resonance Imaging Translational Medical Center, Southwest Hospital, Army Medical University (Third Military Medical University), Chongqing 400038, China; Department of Radiology, Southwest Hospital, Army Medical University (Third Military Medical University), Chongqing 400038, China; 7T Magnetic Resonance Imaging Translational Medical Center, Southwest Hospital, Army Medical University (Third Military Medical University), Chongqing 400038, China; Department of Radiology, Southwest Hospital, Army Medical University (Third Military Medical University), Chongqing 400038, China; 7T Magnetic Resonance Imaging Translational Medical Center, Southwest Hospital, Army Medical University (Third Military Medical University), Chongqing 400038, China; Department of Radiology, Southwest Hospital, Army Medical University (Third Military Medical University), Chongqing 400038, China; Department of Research and Development, Shanghai United Imaging Intelligence Co., Ltd., Shanghai 200232, China; Department of Research and Development, Shanghai United Imaging Intelligence Co., Ltd., Shanghai 200232, China; Department of Research and Development, Shanghai United Imaging Intelligence Co., Ltd., Shanghai 200232, China; MR Research Collaboration Teams, Siemens Healthineers Ltd., Guangzhou 510630, China; 7T Magnetic Resonance Imaging Translational Medical Center, Southwest Hospital, Army Medical University (Third Military Medical University), Chongqing 400038, China; Department of Radiology, Southwest Hospital, Army Medical University (Third Military Medical University), Chongqing 400038, China; 7T Magnetic Resonance Imaging Translational Medical Center, Southwest Hospital, Army Medical University (Third Military Medical University), Chongqing 400038, China; Department of Radiology, Southwest Hospital, Army Medical University (Third Military Medical University), Chongqing 400038, China; 7T Magnetic Resonance Imaging Translational Medical Center, Southwest Hospital, Army Medical University (Third Military Medical University), Chongqing 400038, China; Department of Radiology, Southwest Hospital, Army Medical University (Third Military Medical University), Chongqing 400038, China

**Keywords:** spinocerebellar ataxia type 3, perivascular space, magnetic resonance imaging, correlation analysis

## Abstract

Spinocerebellar ataxia type 3 (SCA3) is an uncommon inherited (autosomal dominant) neurodegenerative disorder caused by abnormal accumulation of ataxin-3 protein. The perivascular space (PVS) burden reflects protein clearance and may worsen in SCA3 disease. This study aimed to quantify the PVS burden and investigate the relationship between the PVS burden and clinical characteristics in individuals with SCA3.

This study enrolled 43 SCA3 patients and 43 age- and sex-matched healthy controls (HCs). The cross-sectional study assessed the severity of ataxia in SCA3 patients using the Scale for the Assessment and Rating of Ataxia (SARA) and the International Cooperative Ataxia Rating Scale (ICARS). Various cognitive functions were evaluated in all subjects using the Montreal Cognitive Assessment (MoCA), Rapid Verbal Retrieval (RAR) and Digital Span Test (DST) scales. MRI was used to automatically segment the PVS in all subjects and quantify the PVS burden in 15 brain regions.

Compared with the HCs, the SCA3 patients showed a significantly higher PVS burden in the basal ganglia, temporal lobe, right parietal lobe and right cerebellum. There was a positive correlation in motor dysfunction between the PVS volume in the left parietal lobe, right cerebellum and PVS number in the right cerebellum with the SARA and ICARS scores.

This study showed that SCA3 patients have an increased PVS burden in many brain regions, leading to motor impairment. The PVS burden could be a new imaging biomarker for disease monitoring and a therapeutic target for SCA3.

## Teaser

Huang *et al*. report that spinocerebellar ataxia type 3 (SCA3) patients exhibit increased perivascular space (PVS) burden in multiple brain regions compared with healthy controls, correlating with motor impairment. The PVS burden may serve as a novel imaging biomarker for SCA3 disease monitoring and a potential therapeutic target.

## Graphical Abstract

**Graphical Abstract:**
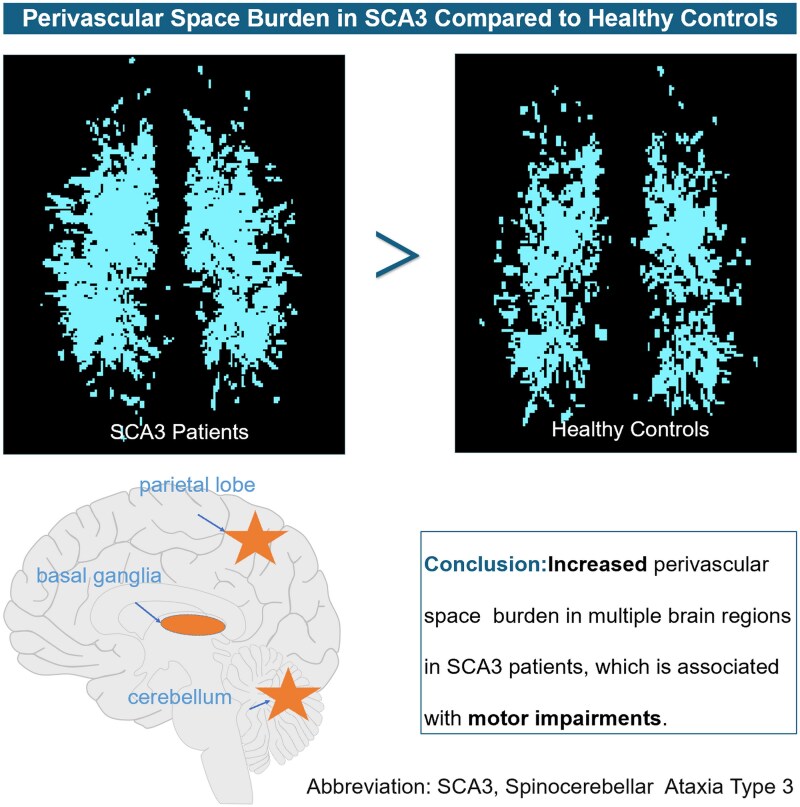

## Introduction

Spinocerebellar ataxia type 3 (SCA3) is a rare inherited (autosomal dominant) neurodegenerative disorder characterized by cerebellum ataxia, dysarthria, disharmony and cognitive impairment.^1^ SCA3 is caused by abnormal amplification of CAG repeats in the *ATXN3* gene.^2^ The subsequent accumulation of the translated ataxin-3 protein in SCA3 results in neuronal damage in the affected regions.^3^ Research involving transgenic mouse models has demonstrated that the accumulation of mutant ataxin-3 proteins can compromise the blood–brain barrier,^4^ resulting in perivascular space (PVS) enlargement.^5^ Thus, an enlarged PVS suggests ataxin-3 protein accumulation and could play an essential role in SCA3 progression.

The PVS constitutes a gap between the two pia mater layers surrounding the perforating blood vessels and passing from the subarachnoid space through the brain tissue.^6^ The PVS enlarges when the cerebrospinal fluid circulation disorder and fluid becomes trapped within the space.^6^ The PVS burden refers to the quantitative assessment of PVS enlargement, encompassing the number of spaces and the volume of fluid contained in the PVS.^7^ In Huntington's disease and Alzheimer's disease, the PVS burden correlates with greater disease severity.^8-11^ Several previous studies have shown that the PVS burden can serve as a biomarker for the onset and progression of neurodegenerative disease.^9,12^ Since we hypothesized that the PVS burden increases in SCA3 patients, correlates with motor impairments and plays a critical role in the progression of SCA3. However, research on the PVS burden in SCA3 is lacking.

Magnetic resonance imaging (MRI) is commonly used to localize and quantify the PVS burden. MRI can dynamically and reliably detect an enlarged PVS non-invasively.^13^ Current methods for analysing the PVS visible with MRI are primarily categorized into visual rating scales and automated segmentation methods.^14^ Visual rating scales are simple and easy to assess PVS burden, but they require manual counting, are time-consuming and lack precision.^15^ In contrast, automated segmentation methods can enhance efficiency and accuracy in PVS assessment and can precisely calculate morphological PVS characteristics,^14,15^ including volume, volume fraction and mean cross-sectional diameter.^16,17^

In this study, we employed automated segmentation with manual quality check methods to extract entire brain PVS masks in SCA3 patients and healthy controls (HCs). We investigated the reliability of the PVS burden as a biomarker. Furthermore, we aimed to explore the association of the PVS burden in SCA3 development and analyse the specific influence mechanism of the PVS on SCA3 progression.

## Materials and methods

### Subjects

**Figure 1:**
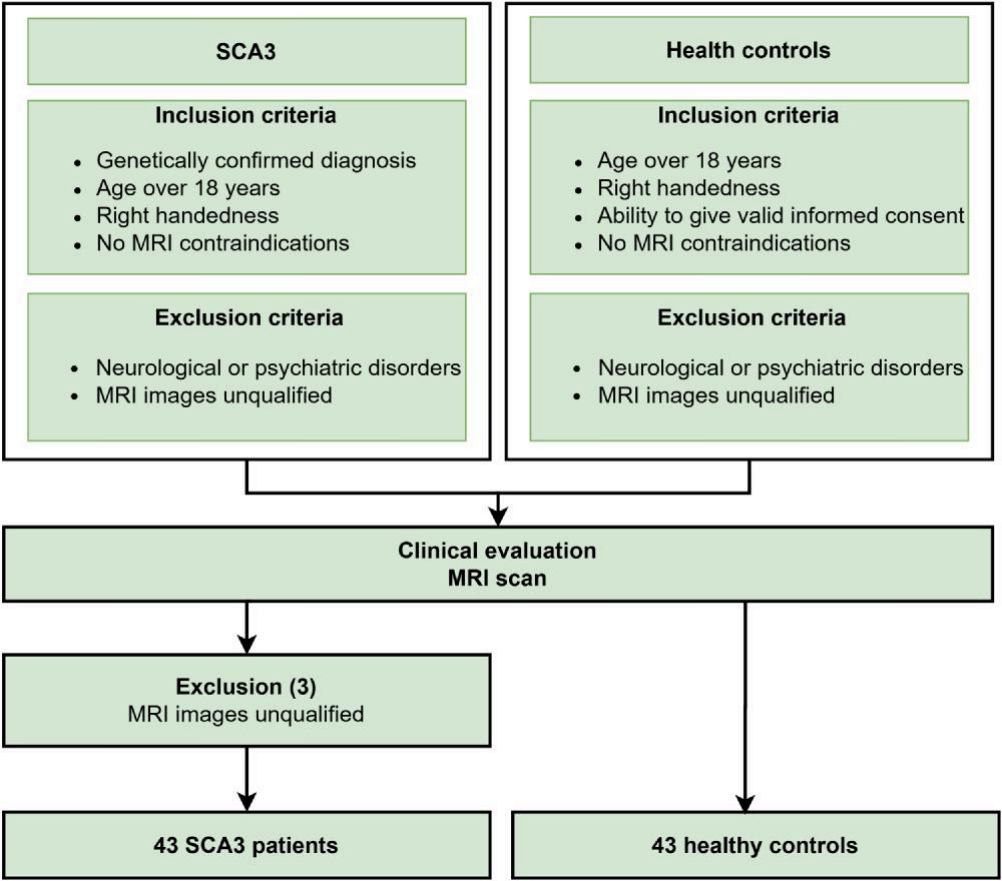
Flowchart of the study subjects.

All subjects were enrolled at the First Affiliated Hospital of Army Medical University from May 2017 to March 2019. The inclusion criteria for the SCA3 participants were a genetically confirmed diagnosis, age over 18 years, right handedness and no contraindications to MRI. The exclusion criteria were other genetic diseases, age less than 18 years, non-right handedness, neurological or psychiatric disorders, contraindications to MRI or unqualified MRI images (*n*  *=* 3). The inclusion criteria for the HCs were age over 18 years, right handedness, no contraindications to MRI and the ability to provide informed consent. The exclusion criteria were age less than 18 years, non-right handedness, neurological or psychiatric disorders, contraindications to MRI or unqualified MRI images (Fig. 1).

Each participant provided informed consent at the First Affiliated Hospital of Army Medical University. The study was approved by the hospital’s Institutional Review Board. The clinical trial registration number for the study is ChiCTR1800019901.

### Clinical evaluation

We assessed ataxia symptom severity in the SCA3 patients using the Scale for the Assessment and Rating of Ataxia (SARA) and the International Cooperative Ataxia Rating Scale (ICARS).^18,19^ All participants were evaluated using neuropsychology. The Montreal Cognitive Assessment (MoCA) scale was used to assess overall cognitive function,^20^ verbal fluency was evaluated with Rapid Verbal Retrieval (RVR) scores and working memory was estimated with the Digital Span Test (DST) test.^21^

### Magnetic resonance imaging

In this study, a 3.0T MAGNETOM Trio scanner (Siemens Healthcare Erlangen, Germany) with phased-array, 8-channel head coil was used for MRI scanning. Subjects were positioned supine with closed eyes during the MRI scan and instructed to remain awake. In addition, foam padding was employed to reduce head movement. Imaging covered the region from the vertex to the foramen magnum. Scanning parameters were acquired using the following scanning protocol:

Sagittal T_1_-weighted images (T1WI): repetition time = 1900 ms, echo time = 2.52 ms, inversion time = 900 ms, echo train length = 1, flip angle = 9°, slice thickness = 1 mm, matrix = 256 × 256, voxel size = 1 × 1 × 1 mm and number of slices = 176.

Axial T_2_-weighted images (T2WI): repetition time = 4490 ms, echo time = 113 ms, inversion time = 720 ms, echo train length = 35, flip angle = 140°, slice thickness = 5 mm, matrix = 220 × 220 and number of slices = 20.

### Image processing

**Figure 2:**
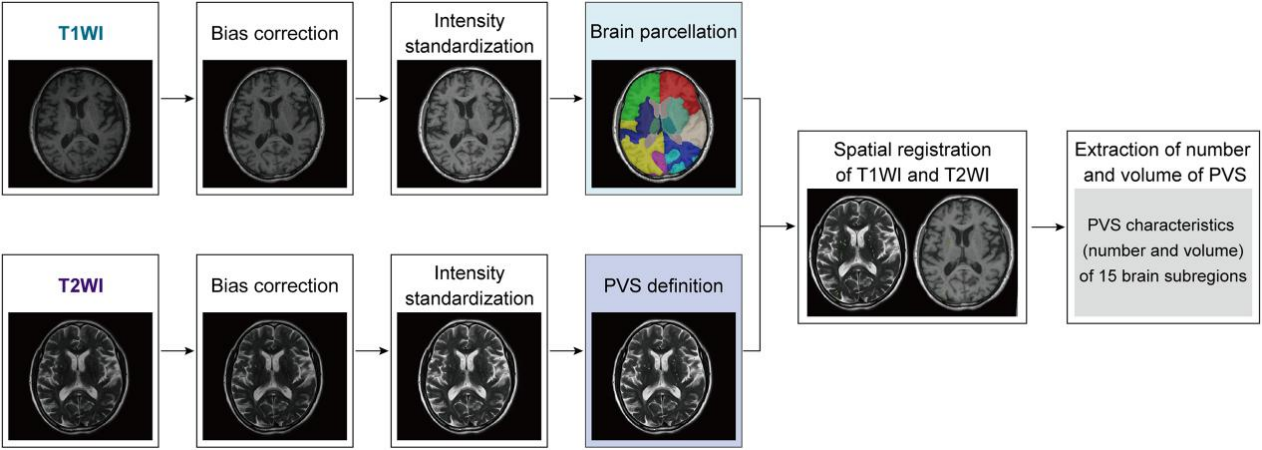
**Schematics showing image processing and methods.** Diagram illustrating the algorithm used to segment the PVS. The progress steps included (i) bias correction, (ii) intensity standardization of T1WI and T2WI, (iii) brain subregions parcellated in T1WI, (iv) PVS definition in T1WI, (v) registration of the PVS mask to brain subregions parcellated in T1WI and (vi) extraction of PVS characteristics of 15 brain subregions.

Image preprocessing comprised several steps (Fig. 2):

1. N4 bias field corrections were applied to both T1WI and T2WI to remove the inhomogeneity of the magnetic field.
2. The greyscale values were standardized, with greyscale intensity values normalized to the range of [−1, 1] by clipping the intensities at 0.1–99.9%.
3. The deep learning model (VB-Net) embedded in the united imaging intelligence artificial intelligence research portal was utilized to remove the skull from T1WI and parcellate the entire brain into 109 regions of interest according to the Desikan-Killiany atlas.^22-25^ These regions were then merged into 15 brain subregions, as detailed in Supplementary Table 1, including bilateral frontal lobes, parietal lobes, occipital lobes, temporal lobes, basal ganglia, cerebellum and thalamus, as well as the brainstem.
4. The PVS was automatically delineated based on T2WI, and the corresponding mask was obtained with a built-in 2D VB-Net model in the uRP platform.^24^ The VB-Net achieved an average dice similarity coefficient greater than 0.90 for PVS. The recall and precision rates for PVS segmentation were over 0.92.^25^
5. The above AI-generated masks were reviewed and revised by two experienced radiologists (C.L. and Y.H.).
6. T1WI and T2WI images were co-registered using a registration algorithm,^26^ and the segmentation mask of the T1WI space was transformed into the T2WI space.
7. The number, volume and density of PVS were quantified for each brain subregion. The PVS density is calculated by determining the average number of tracked PVS points, obtained through the tubular tracking algorithm, within a 2 mm radius of each voxel in the MNI-152 brain template.

### Statistical analysis

Means (standard deviation) were utilized to present normally distributed continuous data, while medians (interquartile range) were used to present non-normally distributed. The frequencies of categorical variables were summarized, and intergroup differences were examined using the chi-squared test. The non-parametric Mann–Whitney U-test assessed differences in the number and volume of PVS between the SCA3 and HCs groups. After adjusting for factors affecting the PVS burden, such as sex, age and total white matter volume,^27^ regression analysis was performed to investigate the correlation between the PVS burden in significant brain regions of the SCA3 patients and their clinical evaluation scale. Statistical analyses were conducted using R statistical software (version 3.6.2) and SPSS software (version 22). Last, graphs were generated with GraphPad Prism software (version 8.0.2). *P*-values less than 0.05 were considered statistically significant.

## Results

### General clinical features

Three SCA3 patients were excluded for MRI images unqualified (Fig. 1), two of them were excluded because of incomplete T2 WI sequence layers and one was excluded because of severe motion artefacts. Forty-three SCA3 patients and 43 HCs were finally enrolled in this study. The demographics and clinical characteristics of all subjects are presented in Table 1. Neither the sex nor age of the groups differed significantly. However, the MoCA (*P*  *<* 0.001), RVR (*P*  *<* 0.001) and DST (*P*  *<* 0.001) scores were significantly lower in the SCA3 patients than in the HCs.

**Table 1:** Demographic and clinical characteristics of the participants

Characteristics	SCA3	HCs	*t*-value	*P*-value
Gender (F/M)	25/18	28/15	0.443	0.506^a^
Age (years)	42.85 ± 11.28	44.00 ± 2.83	−0.441	0.660^b^
Onset age (years)	35.63 ± 10.53	NA		NA
Disease duration(years)	7.4 ± 4.03	NA		NA
CAG repeats	66(64–68)	NA		NA
SARA scores	11.98 ± 8.09	NA		NA
ICARS scores	33.11 ± 21.50	NA		NA
MoCA scores	20.16 ± 8.01	28.58 ± 1.65	−6.746	<0.001^b^
RVR scores	30(25–42)	48(44–56)	6.071	<0.001^c^
DST scores	8(6–8.5)	9(8–10)	3.817	<0.001^c^

NA, not applicable. Data in brackets are interquartile range. Continuous data are presented as mean ± standard deviation or median (Q_2_-Q_3_). The *P*-values are the results of the two-sample *t*-test, Mann–Whitney U-test or *χ*^2^ tests as appropriate between the patients with SCA3 and HCs. ^a^Pearson chi-square test ^b^two-tailed *t*-test and ^c^Mann–Whitney U-test.

### Segmentation performance of VB-Net for perivascular space

After automatic segmentation, the results were manually reviewed, and 6 of 86 cases were manually modified, accounting for 6.9%. Supplementary Fig. 1 shows representative PVS segmentations from sample HC and sample SCA3.

### Comparison of the perivascular space burden between the two groups

**Figure 3:**
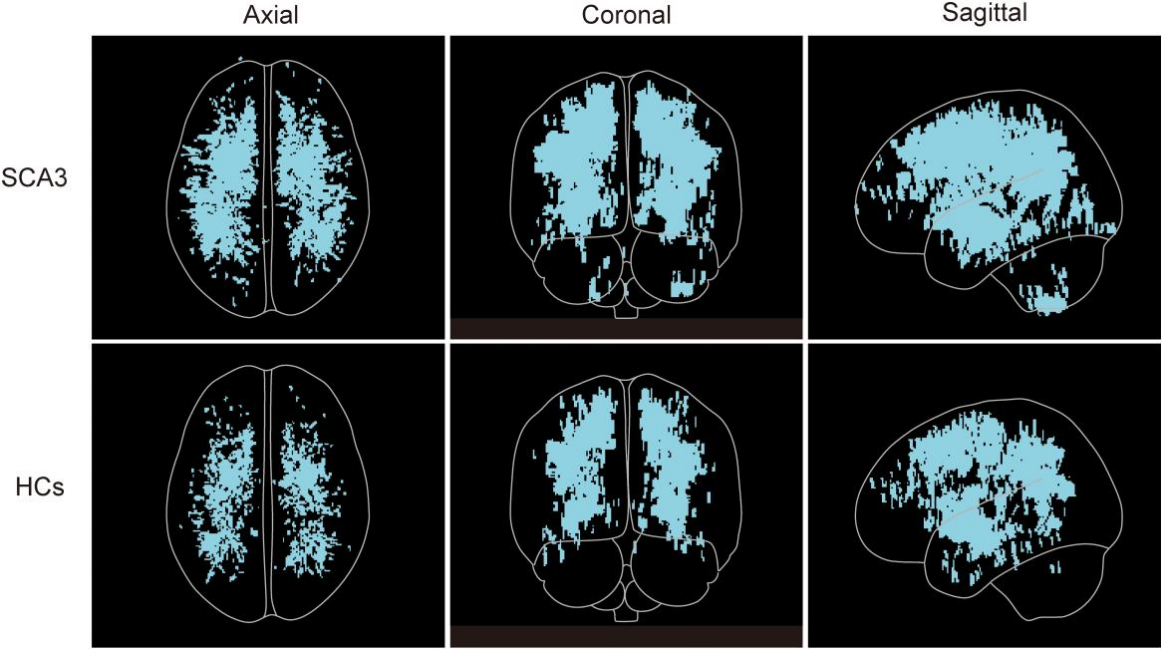
**Maximum intensity projection (MIP) of PVS of SCA3 patients and HCs.** Representative MIP of PVS in axial, coronal and sagittal directions of the entire brain of SCA3 patients (*n*  *=* 43) and HCs (*n*  *=* 43). Upper row: MIP image of PVS in the entire brain of SCA3 patients. Lower row: MIP image of PVS in the entire brain of HCs. MIP images derived from the axial, coronal and sagittal directions are shown, where the number and volume of PVS are higher in the SCA3 patients than in the HCs.

**Figure 4:**
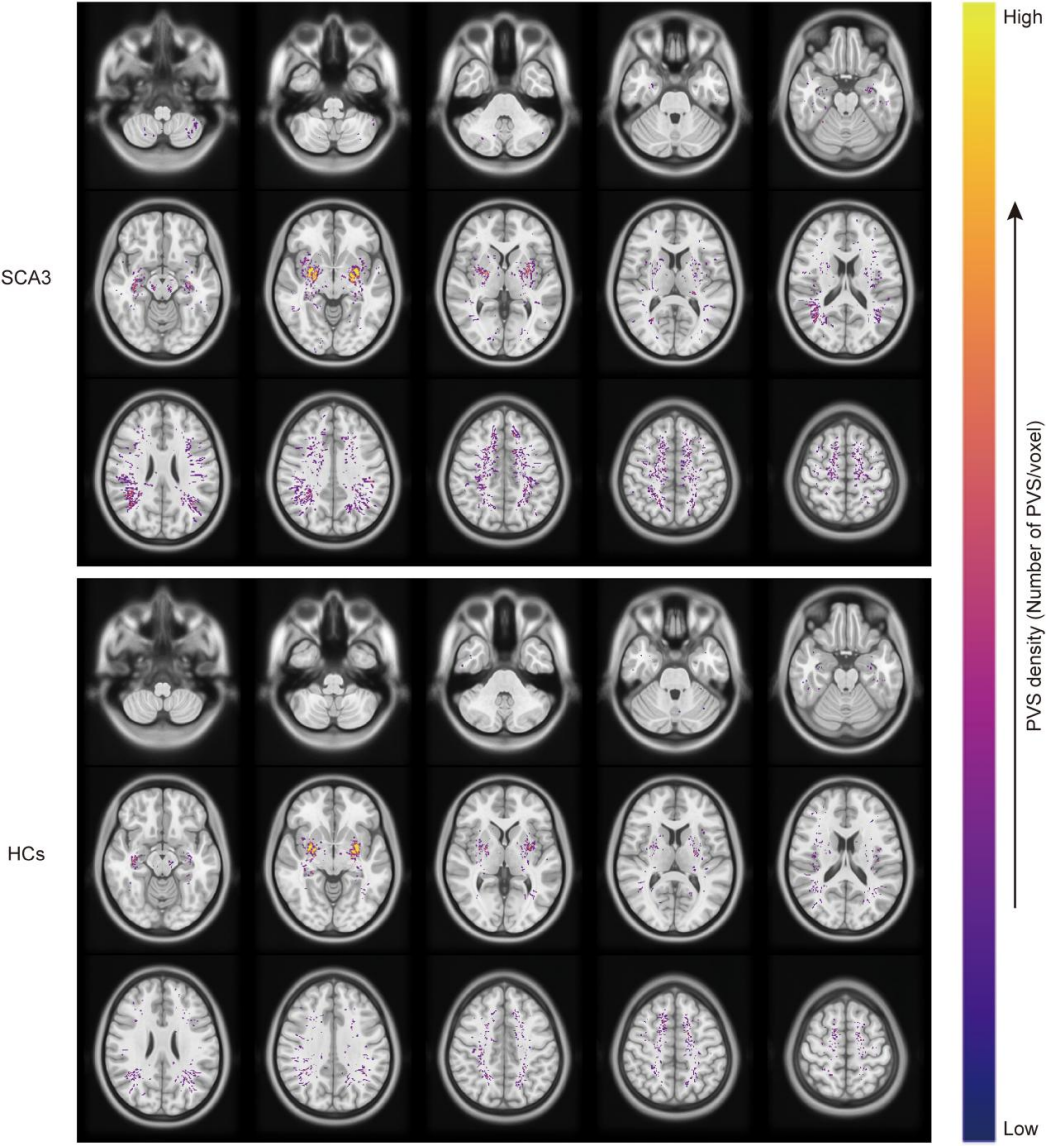
**PVS density is shown in MNI space.** Average distribution of PVS density of the two groups with SCA3 (*n*  *=* 43) and the HCs (*n*  *=* 43), plotted in the Montreal Neurological Institute-152 atlas. The colour indicates the PVS density. PVS densities are similar in both hemispheres in the two groups, with a higher PVS density in the centrum semioval and basal ganglia. Furthermore, a higher PVS density in the SCA3 patients compared with the HCs can be seen.

The study found that the PVS burden in the brains of the SCA3 patients was significantly higher than in the HCs (*P* < 0.001) (Fig. 3). In the SCA3 patients, a higher PVS density was observed in the centrum semioval (*P* < 0.001) and basal ganglia (*P* < 0.001) compare with the HCs, while no significant difference in the PVS density was found between the two hemispheres (*P*  *=* 0.812) (Fig. 4).

**Figure 5:**
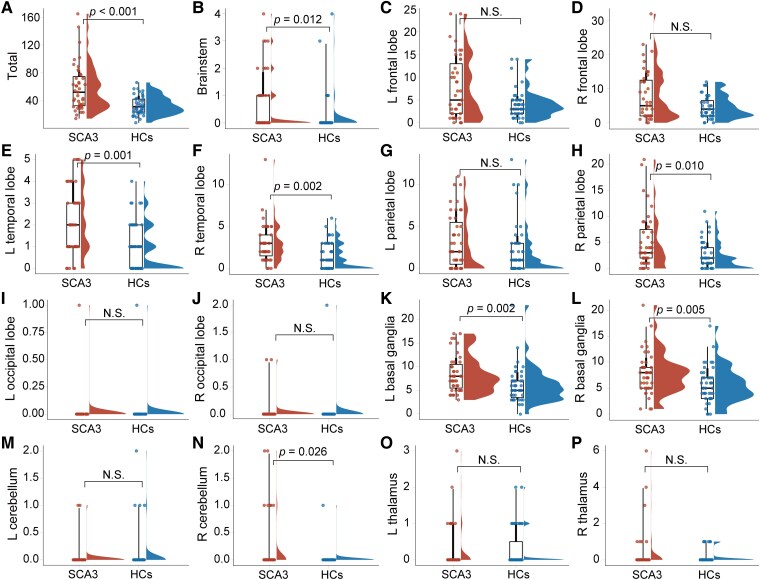
**PVS number comparison between SCA3 patients and HCs.** (**A**) The PVS number of the whole brain, (**B**) the PVS number of the brainstem, (**C**) the PVS number of the left frontal lobe, (**D**) the PVS number of the right frontal lobe, (**E**) the PVS number of the left temporal lobe, (**F**) the PVS number of the right temporal lobe, (**G**) the PVS number of the left parietal lobe, (**H**) the PVS number of the right parietal lobe, (**I**) the PVS number of the left occipital lobe, (**J**) the PVS number of the right occipital lobe, (**K**) the PVS number of the left basal ganglia, (**L**) the PVS number of the right basal ganglia, (**M**) the PVS number of the left cerebellum, (**N**) the PVS number of the right cerebellum, (**O**) the PVS number of the left thalamus and (**P**) the PVS number of the right cerebellum. The ordinate represents the PVS number. Each individual data point represents the PVS number in a specific brain region for an individual subject. Bars represent median (Q_2_-Q_3_). The *P*-values indicate differences between the patients with SCA3 (*n*  *=* 43) and HCs (*n*  *=* 43). The significance level was set at *P*  *<* 0.05. Non-significant (N.S.) *P*  *>* 0.05. The *P-*value was determined by a non-parametric Mann–Whitney U-test and corrected using the Bonferroni method.

Compared with the HCs, the number of PVS in various brain regions was significantly higher in the SCA3 patients. These regions included the left temporal lobes (*P*  *=* 0.001), right temporal lobes (*P*  *=* 0.002), right parietal lobe (*P*  *=* 0.010), left basal ganglia (*P*  *=* 0.002), right basal ganglia (*P*  *=* 0.005), right cerebellum (*P*  *=* 0.026) and brainstem (*P*  *=* 0.012) (Fig. 5).

**Figure 6:**
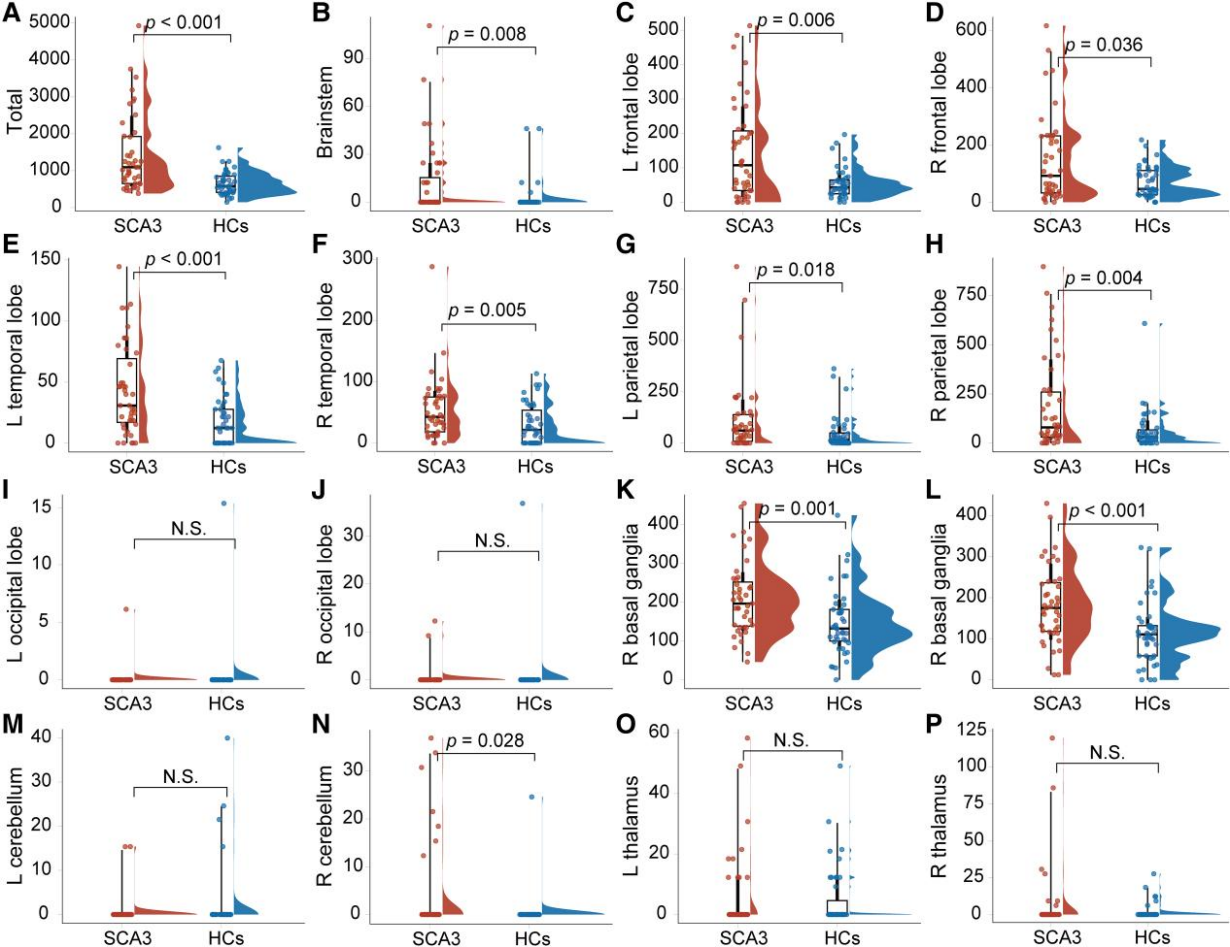
**PVS volume comparison between SCA3 patients and HCs.** (**A**) The PVS volume of the whole brain, (**B**) the PVS volume of the brainstem, (**C**) the PVS volume of the left frontal lobe, (**D**) the PVS volume of the right frontal lobe, (**E**) the PVS volume of the left temporal lobe, (**F**) the PVS volume of the right temporal lobe, (**G**) the PVS volume of the left parietal lobe, (**H**) the PVS volume of the right parietal lobe, (**I**) the PVS volume of the left occipital lobe, (**J**) the PVS volume of the right occipital lobe, (**K**) the PVS volume of the left basal ganglia, (**L**) the PVS volume of the right basal ganglia, (**M**) the PVS volume of the left cerebellum, (**N**) the PVS volume of the right cerebellum, (**O**) the PVS volume of the left thalamus and (**P**) the PVS volume of the right cerebellum. Each individual data point represents the PVS volume in a specific brain region for an individual subject. Bars represent median (Q2-Q3). The ordinate represents the PVS volume, mm^3^. The *P*-values indicate differences between the patients with SCA3 (*n*  *=* 43) and HCs (*n*  *=* 43). The significance level was set at *P*  *<* 0.05. Non-significant (N.S.) *P*  *>* 0.05. The *P*-value was determined by a non-parametric Mann–Whitney U-test and corrected using the Bonferroni method.

Furthermore, this study demonstrated that the SCA3 patients had a significantly higher PVS volume in extensive brain regions than in the HCs. These regions included the left frontal lobes (*P*  *=* 0.006), right frontal lobes (*P=* 0.036), left temporal lobes (*P*  *<* 0.001), right temporal lobes (*P*  *<* 0.005), left parietal lobes (*P*  *=* 0.018), right parietal lobes (*P*  *=* 0.004), left basal ganglia (*P*  *<* 0.001), right basal ganglia (*P*  *=* 0.001), right cerebellum (*P*  *=* 0.026) and brainstem (*P* = 0.008) (Fig. 6).

### Correlation analysis between the perivascular space burden and clinical evaluation scale scores

**Figure 7:**
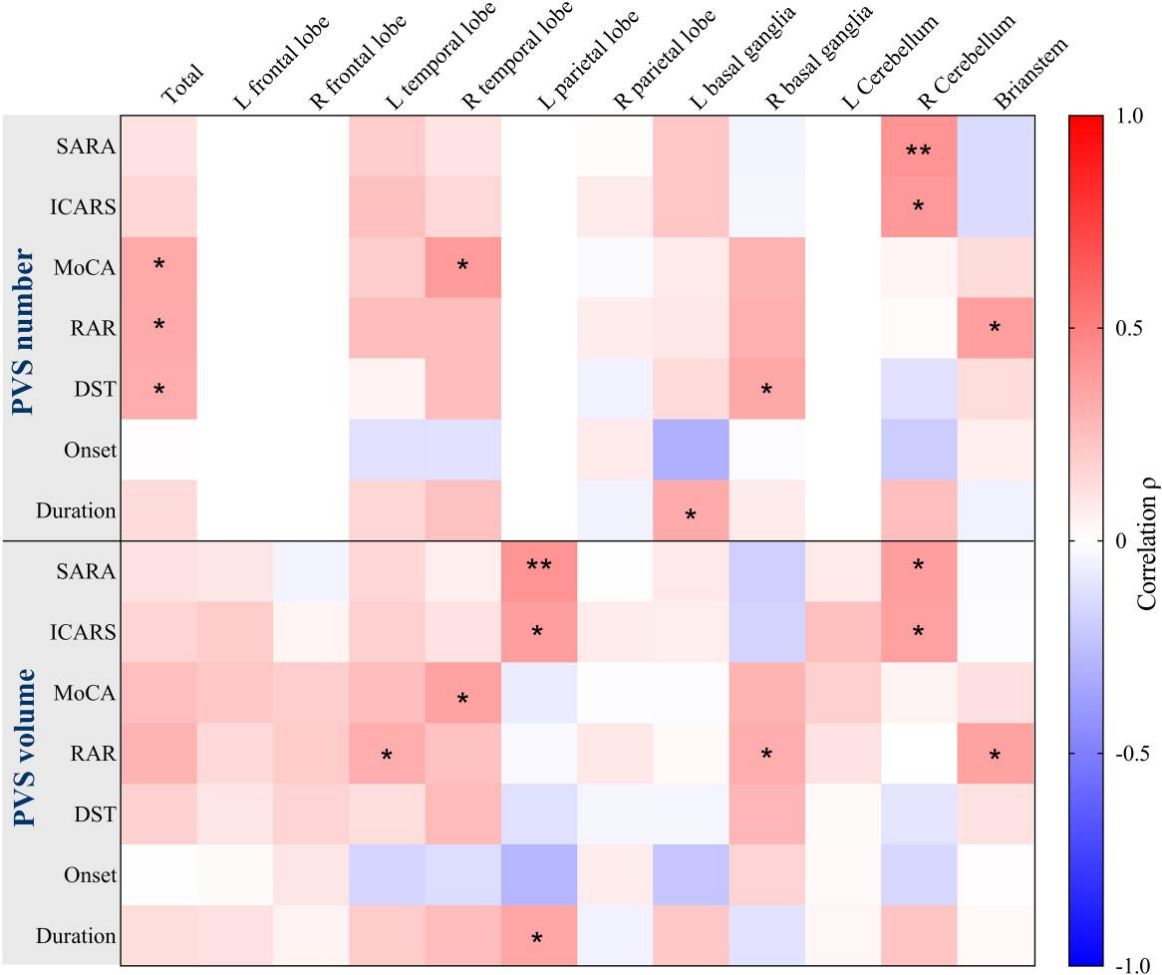
**Correlation analysis between the PVS burden and clinical scale scores in SCA3 patients.** The heatmap displays Spearmen^’^s rank correlation coefficients (*ρ*) between PVS burden metrics (number and volume in specific brain regions) and clinical scores. Analyses were performed in 43 SCA3 patients. The colour scale represents Spearman’s *ρ* ranging from −1 to +1. Asterisks denote significance levels: * *P* < 0.05, ** *P* < 0.01, N.S: *P* > 0.05.

In the correlation analysis between the PVS burden and ataxia rating scale scores, the SARA scores showed a positive correlation with the PVS volume in the left parietal lobe (*r* = 0.416, *P*  *=* 0.008), right cerebellum (*r* = 0.380, *P* *=* 0.016) and PVS number in the right cerebellum (*r* = 0.416, *P*  *=* 0.008). Similarly, the ICARS scores showed a positive correlation with the PVS volume in the left parietal lobe (*r* = 0.378, *P*  *=* 0.016), right cerebellum (*r* = 0.366, *P*  *=* 0.020) and PVS number in the right cerebellum (*r* = 0.397, *P*  *=* 0.011) (Fig. 7).

The correlation analysis between the PVS burden and cognition-related assessment scale scores showed a positive correlation between the MoCA score and the PVS number in the entire brain (*r* = 0.315, *P*  *=* 0.048), right temporal lobe (*r* = 0.382, *P*  *=* 0.015) and PVS volume in the right temporal lobe (*r* = 0.348, *P*  *=* 0.028). In addition, the RAR score showed a positive correlation with the PVS volume in the right basal ganglia (*r* = 0.317, *P*  *=* 0.046), brainstem (*r* = 0.364, *P*  *=* 0.021) and PVS number in the brainstem (*r* = 0.375, *P*  *=* 0.017). Last, the DST score was positively correlated with the PVS number in the entire brain (*r* = 0.328, *P*  *=* 0.039) and right basal ganglia (*r* = 0.341, *P*  *=* 0.031) (Fig. 7).

The duration of disease showed a positive correlation with the PVS volume in the left parietal lobe (*r* = 0.344, *P* *=* 0.030) and PVS number in the left basal ganglia (*r* = 0.329, *P* = 0.038). The age of onset does not have a significant impact on the PVS volume or count in SCA3 patients (Fig. 7).

## Discussion

This study used 3T MR imaging scans and an automated segmentation approach to quantitatively analyse the PVS burden in SCA3 patients and HCs. We explored the association between the PVS burden of different areas of the brain and the clinical evaluation scale scores of SCA3 patients. The principal findings were: (i) The PVS burden in SCA3 patients was significantly higher than in the HCs; (ii) Higher PVS density areas were primarily located in all the subjects’ subcortical white matter and basal ganglia; (iii) There were significant differences in the PVS burden between the two groups primarily in the bilateral frontal, temporal and parietal lobes, basal ganglia, brainstem and right cerebellum (these were mostly volume differences, followed by number differences); (iv) The SARA and ICARS scores in the SCA3 patients were positively correlated with the PVS burden in the right cerebellum and left parietal lobe and (v) The cognitive function assessment scale score of the SCA3 patients was positively associated with the PVS burden, and various correlations were found in different brain regions. Consistent with our hypothesis, the PVS burden in SCA3 patients increased and was associated with severe disease.

In our study, the PVS burden in SCA3 patients was higher than in the healthy individuals. This finding is consistent with the results of previous studies on the PVS burden in patients with other neurodegenerative diseases such as Huntington’s disease and Alzheimer’s disease.^8,9^ The PVS burden in SCA3 patients could be associated with its unique pathological formation mechanism. Studies have shown that the increased PVS burden may induce metabolic waste accumulation, formation of β-amyloid plaques and abnormal protein aggregation in the brain.^9,28,29^ Thus, an increased PVS burden in SCA3 patients may aggravate ataxin-3 protein accumulation, damage the blood–brain barrier and disrupt dynamic cerebrospinal fluid circulation.^29,30^ Conversely, abnormal ataxin-3 protein aggregation, local inflammatory response induced by neuronal necrosis and cerebrospinal fluid circulation disorders result in PVS expansion in SCA3 patients.^2,5,31^ Therefore, the higher PVS burden in SCA3 patients may cause a significant accumulation of ataxin-3 protein as the disease progresses.

In the SCA3 patients, the highest PVS burden areas and density were primarily located in the white matter of the parietal lobes and basal ganglia. The probability of PVS appearing in the white matter region is higher than in the grey matter.^32^ PVS visible on MRI is mostly distributed in areas containing white matter, such as the basal ganglia and centrum semioval.^27^ The regions of higher PVS burden are consistent with extensive atrophy of the brain, basal ganglia, cerebellum and brainstem in SCA3 patients.^33^ However, our study found that the significantly increased PVS burden in the brainstem and right cerebellar regions had a lower PVS density. This finding further suggests that the PVS burden is significant in SCA3 patients. Interestingly, our results showed that the change in the PVS volume in SCA3 patients was more important than the PVS number, consistent with previous studies on multiple sclerosis.^34^ In addition, the finding suggests that in some brain regions, the PVS volume may be a more sensitive indicator than the number, which can change earlier or more efficiently.

Our results showed a significant correlation between motor dysfunction and the increased PVS burden in the left parietal lobe and right cerebellum in SCA3 patients. Studies have demonstrated that the PVS burden in various brain regions is correlated with varied forms of functional impairment in neurodegenerative diseases.^35,36^ The cerebellum and parietal lobe are associated with motor function,^37^ the left parietal lobes help control movement and prepare motor effectors,^38^ and the cerebellum is a complex structure that coordinates motor movements by receiving signals from the brain to ensure precise movement execution.^39^ With the increase in PVS burden, neurons in the left parietal lobe and right cerebellum might be damaged, resulting in blocked signal transmission or disconnection of the neural networks.^40^ This communication disorder between neurons may destroy the correct transmission of motor instructions, affecting patient movement coordination. Furthermore, patients with SCA3 who are right-handed may exhibit lateralization in their dominant brain hemisphere, and this lateralization may exacerbate the condition. Specifically, the left parietal lobe and right cerebellum play a crucial role in processing particular motor tasks, suggesting that lateralization within these regions may have a significant impact on SCA3 symptoms.

A higher PVS burden in the centrum semioval is correlated with worsening memory, and a higher PVS burden in the basal ganglia is correlated with decreased cognitive function in Alzheimer’s disease patients.^41^ Our results showed that the SCA3 patient cognitive function was positively associated with the PVS burden in the right temporal lobe, right basal ganglia and brainstem. This finding contradicts previous studies suggesting that a more significant PVS burden is associated with more severe cognitive impairment.^42,43^ An increased PVS burden may signify diminished clearance of brain waste, potentially leading to adverse effects on cognitive function. Furthermore, multiple factors influence cognition, including white matter hyperintensity, PVS, cerebral microbleeds and lacunar infarcts.^42^ A more comprehensive consideration of influencing factors may be essential to reaching accurate and logical conclusions.

This study had some limitations. First, the small sample size precluded a stratified analysis of SCA3 patients based on disease severity; future studies should have larger sample sizes. Second, the study’s cross-sectional design precluded the evaluation of individual longitudinal changes in PVS burden over time. Including longitudinal data from this cohort in future investigations would provide a better understanding of how this phenomenon develops across individuals. Third, the use of thick slices (5 mm) in our imaging protocol may miss PVS statistics, potentially leading to mischaracterization of PVS continuity and morphology. This limitation arises from the need to balance imaging quality with patient tolerability and clinical feasibility. Future studies should aim to incorporate higher-resolution, thinner-slice imaging to provide a more accurate representation of PVS continuity and address this methodological constraint. Last, our study only demonstrated a correlation analysis between the PVS burden and SCA3 clinical assessment scale scores and did not verify our hypothesis by exploring the underlying mechanism. A future study focusing on foundational research should be conducted to investigate how the PVS burden causes ataxia to progress.

This is the first study to assess the PVS burden in SCA3 patients. We applied the automatic segmentation method to analyse the PVS burden of SCA3 patients quantitatively and explored the correlation between this PVS burden and clinical features. Our study showed that the PVS burden is significantly higher in the SCA3 patients compared with the HCs and that the PVS burden is correlated with motor and cognitive dysfunction in SCA3 patients. Our results confirm the PVS burden’s critical role in the SCA3 process, proposing a novel imaging biomarker for disease monitoring and establishing a potential theoretical foundation for future treatments.

## Supplementary Material

fcaf118_Supplementary_Data

## Data Availability

The datasets used and/or analysed during the current study are available from the corresponding author upon reasonable request. The R code used for statistical analysis is also available upon request.
