# Supplementary material for: Beyond the cerebellum: perivascular space burden in spinocerebellar ataxia type 3 extends to multiple brain regions
Source: Brain Commun. 2025 Mar 27;7(2):fcaf118. doi: 10.1093/braincomms/fcaf118 (PMC11969673; doi:10.1093/braincomms/fcaf118)
Supplement: fcaf118_Supplementary_Data [file fcaf118_supplementary_data.docx]

| Number | Brain subregions | Anatomical structures |
| --- | --- | --- |
| 1 | Frontal_Lobe_L | Precentral_L, Frontal_Sup_L, Frontal_Mid_Rostral_L, Frontal_Mid_Caudal_L, Frontalpole_L, Orbitofrontal_Lat_L, Orbitofrontal_Med_L, Parsopercularis_L, Parsorbitalis_L, Parstriangularis_L |
| 2 | Frontal_Lobe_R | Precentral_R, Frontal_Sup_R, Frontal_Mid_Rostral_R, Frontal_Mid_Caudal_R, Frontalpole_R, Orbitofrontal_Lat_R, Orbitofrontal_Med_R, Parsopercularis_R, Parsorbitalis_R, Parstriangularis_R |
| 3 | Parietal_Lobe_L | Postcentral_L, Paracentral_L, Parietal_Sup_L, Parietal_Inf_L, Precuneus_L, Supramarginal_L |
| 4 | Parietal_Lobe_R | Postcentral_R, Paracentral_R, Parietal_Sup_R, Parietal_Inf_R, Precuneus_R, Supramarginal_R |
| 5 | Occipital_Lobe_L | Cuneus_L, Lingual_L, Pericalcarine_L, Occipital_Lat_L |
| 6 | Occipital_Lobe_R | Cuneus_R, Lingual_R, Pericalcarine_R, Occipital_Lat_R |
| 7 | Temporal_Lobe_L | Hippocampus_L, Parahippocampal_L, Entorhinal_L, Fusiform_L, Temporal_Sup_L, Temporal_Mid_L, Temporal_Inf_L, Temporalpole_L, Temporal_Sup_Banks_L, Transversetemporal_L |
| 8 | Temporal_Lobe_R | Hippocampus_R, Parahippocampal_R, Entorhinal_R, Fusiform_R, Temporal_Sup_R, Temporal_Mid_R, Temporal_Inf_R, Temporalpole_R, Temporal_Sup_Banks_R, Transversetemporal_R |
| 9 | Cerebellum_L | Cerebellum_Cortex_L, Cerebellum_WM_L |
| 10 | Cerebellum_R | Cerebellum_Cortex_R, Cerebellum_WM_R |
| 11 | Thalamus_L | Thalamus_L, VentralDC_L |
| 12 | Thalamus_R | Thalamus_R, VentralDC_R |
| 13 | Basal_Ganglia_L | Amygdala_L, Caudate_L, Putamen_L, Pallidum_L, Accumbens_Area_L, Insula_L |
| 14 | Basal_Ganglia_R | Amygdala_R, Caudate_R, Putamen_R, Pallidum_R, Accumbens_Area_R, Insula_R |
| 15 | Brainstem | Pons, Midbrain, Medulla, SCP |

**Supplementary Table 1. Brain subregions details**

L, left; R, right. A total of 15 brain subregions were obtained by automatic brain parcellation and merging neighboring anatomical structures. Other anatomical structures, including Cerebral_WM, Corpus_Callosum, and Cingulate_Gyrus, were divided into frontal, parietal, occipital, and temporal lobes according to the principle of proximity.


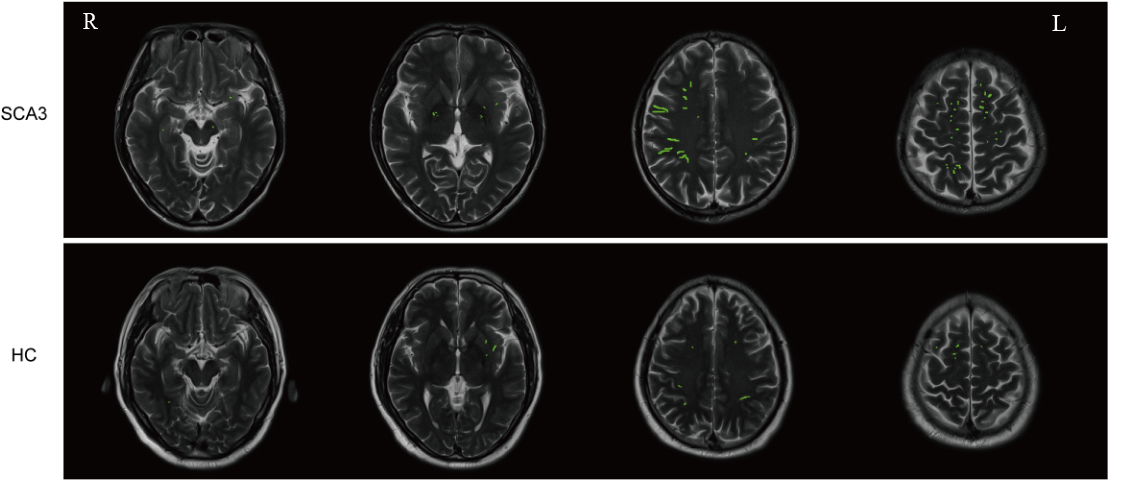


**Supplementary Figure 1. Representative PVS segmentations from a sample SCA3 and sample HC.**

Axial T2-weighted MRI slices with green overlays indicating automatically segmented PVS using the VB-Net model. The segmentation results were manually reviewed and refined by two radiologists. L (left) and R (right) labels denote hemispheric orientation (neurological convention). The experimental unit (*n*) for this figure is one SCA3 patient and one HC, selected as representative examples from the total cohort (*n*=86: 43 SCA3, 43 HCs). The VB-Net model achieved a mean dice similarity coefficient >0.90 and precision/recall rates >0.92 for PVS segmentation.
